# Viscosity dictates metabolic activity of *Vibrio ruber*

**DOI:** 10.3389/fmicb.2012.00255

**Published:** 2012-07-18

**Authors:** Maja Borić, Tjaša Danevčič, David Stopar

**Affiliations:** Chair of Microbiology, Biotechnical Faculty, Department of Food Science and Technology, University of LjubljanaLjubljana, Slovenia

**Keywords:** viscosity, physiology, metabolism, *Vibrio*, prodigiosin

## Abstract

Little is known about metabolic activity of bacteria, when viscosity of their environment changes. In this work, bacterial metabolic activity in media with viscosity ranging from 0.8 to 29.4 mPas was studied. Viscosities up to 2.4 mPas did not affect metabolic activity of *Vibrio ruber*. On the other hand, at 29.4 mPas respiration rate and total dehydrogenase activity increased 8 and 4-fold, respectively. The activity of glucose-6-phosphate dehydrogenase (GPD) increased up to 13-fold at higher viscosities. However, intensified metabolic activity did not result in faster growth rate. Increased viscosity delayed the onset as well as the duration of biosynthesis of prodigiosin. As an adaptation to viscous environment *V. ruber* increased metabolic flux through the pentose phosphate pathway and reduced synthesis of a secondary metabolite. In addition, *V. ruber* was able to modify the viscosity of its environment.

## Introduction

Bacteria are usually studied as planktonic cells in liquid media of low viscosity or as individuals integrated into a highly structured viscous environment of biofilms. Bacterial gene expression and behavior greatly varies between these two lifestyles (Davey and O'Toole, 2000; Walters et al., 2003; Fux et al., 2005; Resch et al., 2005, 2006; Spormann, 2008; Stewart and Franklin, 2008). Liquid media of low viscosity and structured biofilms represent two extremes that leave out many intermediate viscosity environments such as human mucus, tears, saliva and ovulatory mucus (Cone, 1999; Lai et al., 2009). Little is known about how bacteria alter metabolism in environments with intermediate viscosity.

Being a fundamental property of liquids, viscosity plays an important role in fluid flow, molecular diffusion and transport processes (Kao et al., 1993; Swaminathan et al., 1996). Much less is known about the effect of viscosity on metabolic activity of bacteria. In highly viscous biofilm environment for example, the flow of nutrients and O_2_ is changed, leading to metabolite accumulation and gradient formations, which presumably cause differences in bacterial physiology (Davey and O'Toole, 2000; Walters et al., 2003; Fux et al., 2005; Resch et al., 2005, 2006; Spormann, 2008; Stewart and Franklin, 2008). Consequently, the pattern of gene expression is significantly different (Resch et al., 2005, 2006; Folsom et al., 2010). It has been reported that the overall metabolic activity of cells in naturally occurring biofilms is lower than in planktonic cultures (Zheng and Stewart, 2004; Folsom et al., 2010). However, it is important to note that growth rates of biofilm cells are lower (Svensäter et al., 2001; Folsom et al., 2010). Viscosity markedly affects bacterial motility and at high viscosities cells become immobilized (Schneider and Doetsch, 1974; Greenberg and Canale-Parola, 1977; Ferrero and Lee, 1988). For example, short rods or cocci experience impaired motility in highly viscous environments compared to long, curved bacteria like *Campylobacter jejuni* or spirochaetes (Shoesmith, 1960; Schneider and Doetsch, 1974; Ferrero and Lee, 1988; Shigematsu et al., 1998; Nakamura et al., 2006; Swidsinski et al., 2007). In addition, the flagellation type of bacterial cells significantly influences their motility. For instance, at viscosities up to 60 mPas bacteria with polar flagellum swim noticeably slower than laterally flagellated bacteria (Schneider and Doetsch, 1974; Greenberg and Canale-Parola, 1977; Ferrero and Lee, 1988).

Many important collective bacterial activities like biofilm formation are often mediated by small molecules secreted and sensed by cells (Davies et al., 1998; Parsek and Greenberg, 2000; Hammer and Bassler, 2003; Shrout et al., 2011). Such communication between cells has been found in various bacterial species and is thought to enable cooperative coordination and regulation of gene expression for traits that might confer group benefits (Schauder and Bassler, 2001) such as virulence, production of exoenzymes and extracellular polymers (Davies et al., 1998; Miller et al., 2002; Sakuragi and Kolter, 2007; Williams et al., 2007). The triggering of these processes relies on the threshold concentration of signals in the medium and is dependent on diffusion (Redfield, 2002; Hense et al., 2007; Horswill et al., 2007). It has been shown that the concentration of signaling molecules falls off sharply with increasing distances from the producer (Alberghini et al., 2009). The gradient is expected to be even more pronounced with increasing viscosity, which reduces diffusion and may therefore decrease signaling efficiency at higher viscosities.

In this study, a simple model system was developed in which viscosity of the growth medium was gradually increased. Viscosity of the minimal M9 medium was experimentally manipulated with a thickening agent, hydroxyethyl cellulose (HEC) that could not be used as a sole carbon source by *Vibrio ruber* DSM 14379. *V. ruber* was isolated from the coastal estuarine environment with regular summer microbial blooms. In its environment it is subjected to changes in viscosity, not only due to temperature changes (Fofonoff, 1962), but also due to presence of microbial polysaccharides released during microbial blooms, which increase local viscosity of the environment. In addition, vibrios are able to form viscous biofilm structures (Yildiz and Visick, 2008). Viscosity was increased up to 29.4 mPas, which is intermediary between the viscosity of the minimal growth medium (0.8 mPas) and viscosity of biofilms [i.e., 1 Pas or more (Hall-Stoodley et al., 2004; Cheong et al., 2009)]. Bacterial physiological status was determined by growth rate, intracellular metabolic activity (cell respiration, dehydrogenase activity, and activity of glycolytic enzymes), synthesis of extracellular polymeric substances (EPS), and prodigiosin production. The latter is controlled by intercellular communication (Thomson et al., 2000; Danevčič and Stopar, 2009). The red pigment prodigiosin is a secondary metabolite that has antimicrobial, immunosuppressive, and anticancer activity (Pérez-Tomás et al., 2003; Williamson et al., 2006). Its antimicrobial properties might provide *V. ruber* a competitive advantage in the environment (Starič et al., 2010). It has been suggested that in bacterial cells, prodigiosin might have a role in energy spilling reaction (Haddix et al., 2008), it may function as a metabolic sink for NAD(P)H or proline (Hood et al., 1992), or as an anion exchanger (Seganish and Davis, 2005). Additionally, it is important in bacterial air dispersal (Burger and Bennett, 1985), storage of light energy (Ryazantseva et al., 1995), and UV survival (Borić et al., 2011).

## Materials and methods

### Bacterial strain, gene sequencing, and phylogenetic analysis

**Figure A1:**
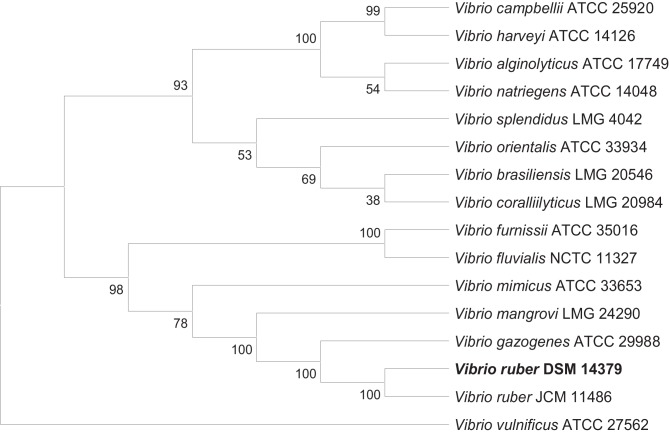
**Phylogenetic tree based on the Maximum Parsimony (MP) method using concatenated sequences (4675 nucleotides) of 16S rRNA, *rpoA*, *recA*, *ftsZ*, *gapA*, *gyrB*, and *mreB* genes of 16 closely related *Vibrio* species.** Bootstrap percentages after 1000 replications are shown next to the branches. Tree length is 3598.

The bacterial strain used in this study was isolated and characterised as described previously (Stopar et al., 2004; Borić et al., 2011) and was designated as *Vibrio* sp. DSM 14379 in our previous publications. In order to determine the strain more precisely, we have performed further genetic analysis. Seven gene loci (16S *rRNA*, *rpoA*, *recA*, *ftsZ*, *gapA*, *gyrB*, and *mre*B) of 15 closely related *Vibrio* type strains were chosen to construct a phylogenetic tree. The accession numbers for the sequences used in Multilocus Sequence Analysis (MLSA) to construct the phylogenetic tree are shown in Table A1. The results show that our strain is closely related to *Vibrio ruber* JCM 11486 (Figure A1). According to the MLSA data, we have named the strain *Vibrio ruber* DSM 14379 and deposited the strain in DSM.

### Bacterial growth

Minimal M9 medium (10.2 g Na_2_HPO_4_, 3 g KH_2_PO_4_, 1 g NH_4_Cl, 2 mL 1 M MgSO_4_·7H_2_O, and 0.1 mL 1 M CaCl_2_·2H_2_O per litre) supplemented with 30 g NaCl and 10 g/L glucose (Starič et al., 2010) was used to grow *V. ruber*. Although glycerol is an obvious choice for medium viscosity manipulation it was not used in this study, because *V. ruber* is able to metabolize it and use it as a sole carbon source. In addition, various polymers that are known to change viscosity have been tested, but most of them were degraded by *V. ruber*. HEC was selected, because *V. ruber* was not able to use it as a sole carbon source. No growth was observed in M9 medium with HEC (Ashland, North Carolina, USA) after two weeks of incubation at 28°C on an orbital shaker at 200 rpm in the dark. Cellulolytic activity was assessed according to Miller (1959) in M9 medium with 1% (w/V) HEC with and without 10 g/L glucose. *V. ruber* cultures were sampled at the time of inoculation and after 24 h of incubation at 28°C and 200 rpm and reducing sugar concentration was determined. In viscosity experiments HEC was added to growth media in various concentrations [i.e., 0.1, 0.25, 0.5, and 1% (w/V)]. The corresponding viscosities were 1.3, 2.4, 8.1, and 29.4 mPas. Concentrations of dissolved oxygen in growth media supplemented with HEC were measured with Oakton PCD 650 multimeter (Oakton Instruments, Illinois, USA). The water activity, *a*_*w*_, of growth media with different HEC concentrations was measured with the CX-1 system (Campbell Scientific Ltd.) according to manufacturer instructions.

M9 growth medium with an appropriate HEC concentration was inoculated with 1% (V/V) of an overnight bacterial culture and incubated at 28°C, in the dark, on an orbital shaker at 200 rpm with orbit length of 20 mm. Optical density at 650 nm was measured spectrophotometrically at regular time intervals. Growth rates of *V. ruber* cultures in M9 media with different viscosities were determined from growth curves with a logistic equation according to Danevčič et al. (2005). Bacterial cell numbers were determined by colony forming units (CFU) counting. Cell size was measured under the inverted microscope Axio Observer Z1 (Carl Zeiss, Germany) using AxioVision 4.8 program. Bacterial flagellation was observed under transmission electron microscope (TEM) Philips CM100 (Philips Electronics N.V., The Netherlands). Bacterial cells were negatively stained with 1% (V/V) uranyl acetate.

### Viscosity measurements and sample preparation

Viscosity was measured on Anton Paar Physica MCR 301 rotational rheometer (Anton Paar, Graz, Austria). The plate-plate system was used with a plate diameter of 50 mm, distance between plates was 0.25 mm and the measuring temperature was (25.00 ± 0.01)°C. Approximately 750 μL of sample was applied to fill the gap between the plates. Flow curves in a shear rate ranging from 1 to 1000 s^−1^ were measured in 29 steps with a time delay of 5 s between successive measurements. Unless stated otherwise, the results of viscosity measurements are reported at a shear rate of 1000 s^−1^.

To determine the effect of conditioned medium on viscosity, 1% (V/V) of overnight *V. ruber* culture was transferred into M9 medium with 10 g/L glucose and 1% (w/V) HEC and incubated for 1 h at 28°C and 200 rpm in the dark. Supernatant was collected by centrifugation at 9391 *g* for 10 min and then incubated either at room temperature or 100°C for 15 min. Incubation at 100°C was used to inactivate cellulolytic enzymes. Both samples were mixed in 1:1 (V/V) ratio with sterile M9 medium without HEC. Viscosity of these samples was measured immediately after the addition of the fresh medium and after 24 h of incubation at 28°C. As a control, sterile distilled water was mixed with M9 medium containing 10 g/L glucose and 1% (w/V) HEC in 1:1 (V/V) ratio.

### Extraction of extracellular polymeric substances

*V. ruber* was grown in the M9 medium containing 10 g/L glucose with an appropriate HEC concentration to the late exponential growth phase at 28°C and 200 rpm. 10 mL of bacterial culture were mixed with 10 mL of phosphate buffer (8 g NaCl, 0.2 g KCl, 1.44 g Na_2_HPO_4_, 0.24 g KH_2_PO_4_ per liter of distilled water pH 7). The mixture was transferred into eppendorf tubes and sonicated (5 s with ultrasound amplitude 12 μm and power 3.5 W/cm^2^) to release free EPS. To isolate cell bound EPS 1 M NaOH was added to the mixture to the final concentration of 0.1 M (Li et al., 2002), shortly vortexed and incubated for 5 min at room temperature. The samples were again shortly vortexed and incubated on ice for 5 min to cool down before adding cold 1 M HCl to the final concentration of 0.2 M. The pH of the samples after HCl addition was around 1.7, which avoids salt and HEC precipitation from the media. The samples were centrifuged at 10,397 g for 15 min at 4°C to remove cells. An aliquot of the supernatant was transferred into a three volumes of cold 96% ethanol (D'Abzac et al., 2010) and incubated at 4°C for 20 h to precipitate EPS. After incubation, the precipitated EPS was collected by centrifugation at 10,397 g for 10 min at 4°C and re-dissolved in distilled water using the volume, which was equivalent to ten volumes of the pellet. The EPS was then re-precipitated by transferring the re-dissolved pellet into a three volumes of cold 96% ethanol, followed by the incubation at 4°C for 20 h. Finally, EPS was collected by centrifugation at 10,397 g for 10 min at 4°C and dried at 105°C. The dried EPS was weighted to calculate the amount of EPS produced per cell. HEC was not precipitated during the extraction procedure. The number of viable cells was determined by CFU counts.

### Cell respiration and dehydrogenase activity

Respiration rate was determined as described previously (Odić et al., 2007; Danevčič and Stopar, 2011). Briefly, 5 mL of bacterial culture was centrifuged for 15 min at 14,972 *g* and 4°C. Cells were washed and resuspended in 5 ml of 3% (w/V) NaCl solution. Bacterial cell suspension was transferred into air tight sterile serum bottles. The ratio between the gas and liquid phase was 2:1 (V/V). The amount of released CO_2_ was measured on a gas chromatograph with thermal conductivity detector (TCD) at the beginning and after one hour of incubation at 28°C and 150 rpm (Odić et al., 2007). Control samples contained 5 mL of 3% (w/V) NaCl solution. The number of viable cells was determined for every sample by CFU counts. Specific cell respiration is given as millilitres of CO_2_ produced per hour, per cell. Relative respiration rate was obtained with normalization to the respiration rate measured at the lowest viscosity (0.8 mPas).

Dehydrogenase activity of *Vibrio* sp. DSM 14379 was determined as described previously (Danevčič and Stopar, 2011). Briefly, 10 mL of bacterial culture was centrifuged at 14972 *g* for 15 min at 4°C. Cells were washed, resuspended and vortexed in 20 mM Tris-HCl buffer supplied with 3% (w/V) NaCl. Next, one mL of 1% (w/V) TTC in 0.1 M Tris-HCl (pH 7.7), 50 μL of 0.5 M KH_2_PO_4_, and 50 μL of 1 M glucose were added to the mixture. No TTC was added to the control samples. Samples were incubated in the dark for one hour, on an orbital shaker at 100 rpm and 28°C to allow TTF formation. TTF was extracted from cells with methanol and its concentration was determined spectrophotometrically at 485 nm. Protein content was determined with Bradford reagent (Sigma, USA) according to manufacturer instructions. Dehydrogenase activity is given as μmol of TTF produced per minute (units) per mg cell protein. Relative dehydrogenase activity was normalized to dehydrogenase activity measured at the lowest viscosity (0.8 mPas).

### Activity of glycolytic enzymes

*V. ruber* was grown in the M9 medium containing 10 g/L glucose with an appropriate HEC concentration to the late exponential growth phase at 28°C and 200 rpm. Cells were harvested by centrifugation at 14,972 *g* for 15 min at 4°C and then washed in a 3% (w/V) NaCl solution. Cell extracts were prepared according to Danevčič and Stopar (2011). Briefly, cell pellets were concentrated 300-fold and sonicated 12 times for 30 s with ultrasound amplitude 6 μm and power 3.5 W/cm^2^ to release intracellular enzymes from bacterial cells. Cell extracts were obtained by removing cell debris with centrifugation at 10,397 g for 10 min at 4°C. Pyruvate kinase (PK) and glucose-6-phosphate dehydrogenase (GPD) activity were determined in cell extracts according to Padilla et al. (2004). In PK assay 1 mM NADH was used instead of 3 mM NADH as originally described. Phosphofructokinase (PFK) activity was determined according to Andersen et al. (2001), the final NADH concentration was 0.5 mM NADH instead of 0.2 mM used in the original protocol. The kinetics of all three glycolytic enzymes was measured either by production or consumption of NADH. The absorbance of NADH was measured at 340 nm and change of A_340_ against time was recorded at 28°C. The slope of the linear part of the kinetic curves represents the rate of enzymatic reaction. Protein content in cell extracts was determined with Bradford reagent (Sigma, USA) according to manufacturer instructions. The results for enzyme activity were calculated according to Danevčič and Stopar (2011) in U per mg cell protein, and are given as relative enzyme activities normalized to the enzyme activity measured at the lowest viscosity (0.8 mPas).

### Prodigiosin production

Prodigiosin produced by *V. ruber* was extracted with acetone as described by Borić et al. (2011). Absorption spectra were measured in 300 μL of extracts using THERMO Multiscan Spectrum (Thermo Electron Company, Vantaa, Finland) at room temperature in a wavelength range from 380 to 600 nm. The obtained spectra were corrected for acetone background absorption and the amount of prodigiosin was determined as described previously (Starič et al., 2010). HEC used for viscosity manipulation did not affect pigment extraction. The data for prodigiosin production during growth were fitted with Boltzmann fit (R^2^ of all fits were above 0.997). The first derivatives of the Boltzmann fits were plotted against time and were approximated with Gaussian distribution (R^2^ > 0.998). Two parameters of Gaussian distribution were used to describe pigment production dynamics—width of the distribution (*W*) representing the duration of intense pigment production and the peak of the Gauss curve (*X*_*c*_) representing the time of maximal pigment production.

### Statistical analysis

All results were statistically analyzed with two sample Student's *t*-test. Differences with *p*-values ≤ 0.05 were considered statistically significant.

## Results

### Viscosity and EPS production

**Figure 1:**
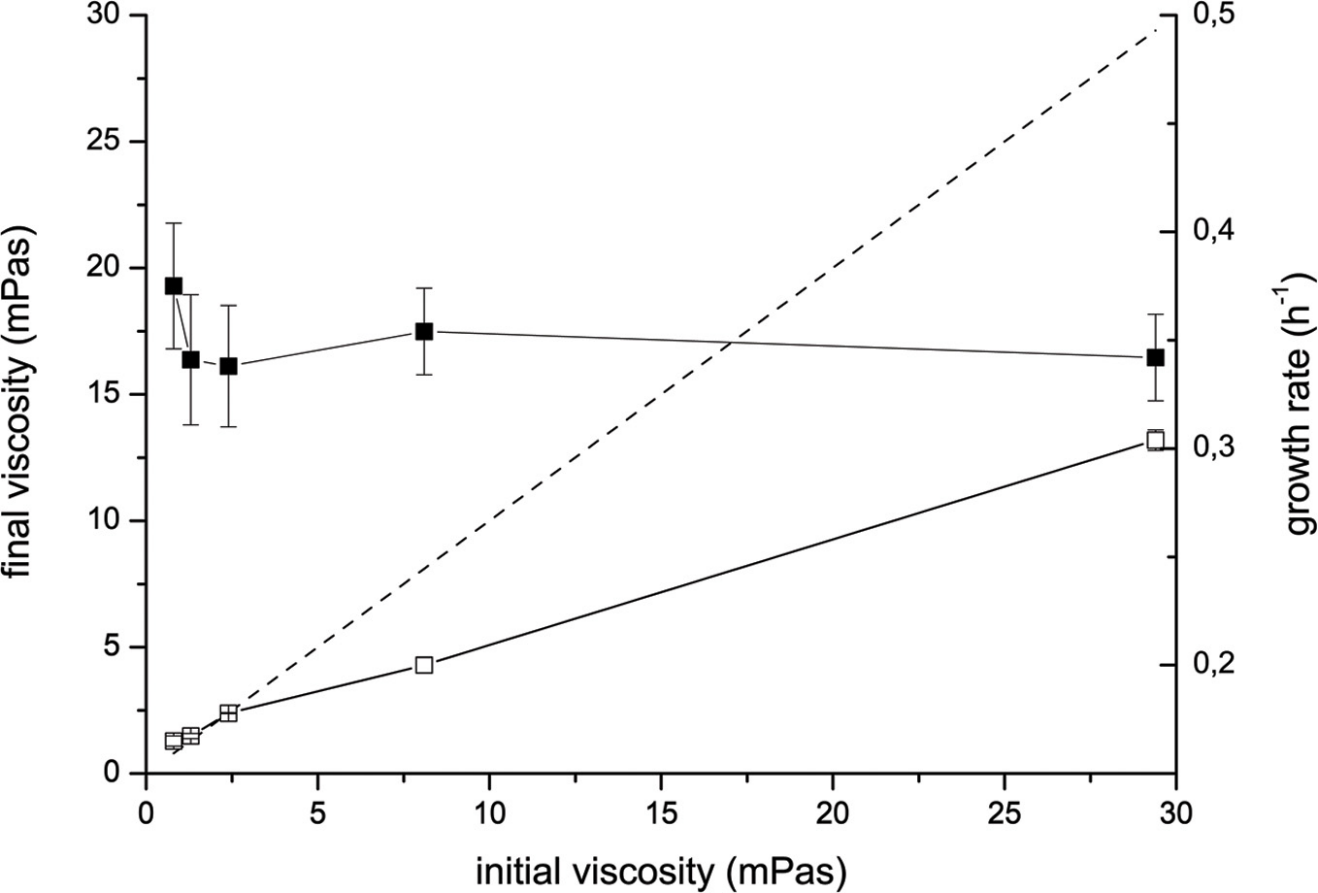
**Changes of viscosity of the M9 medium supplemented with HEC incubated with *V. ruber* (open symbols) and without *V. ruber* (dashed line).** Viscosities were measured at the beginning and at the end of the incubation. Filled symbols represent the growth rate of *V. ruber*. The values presented are means and standard deviations (*n* ≥ 3).

**Figure 2:**
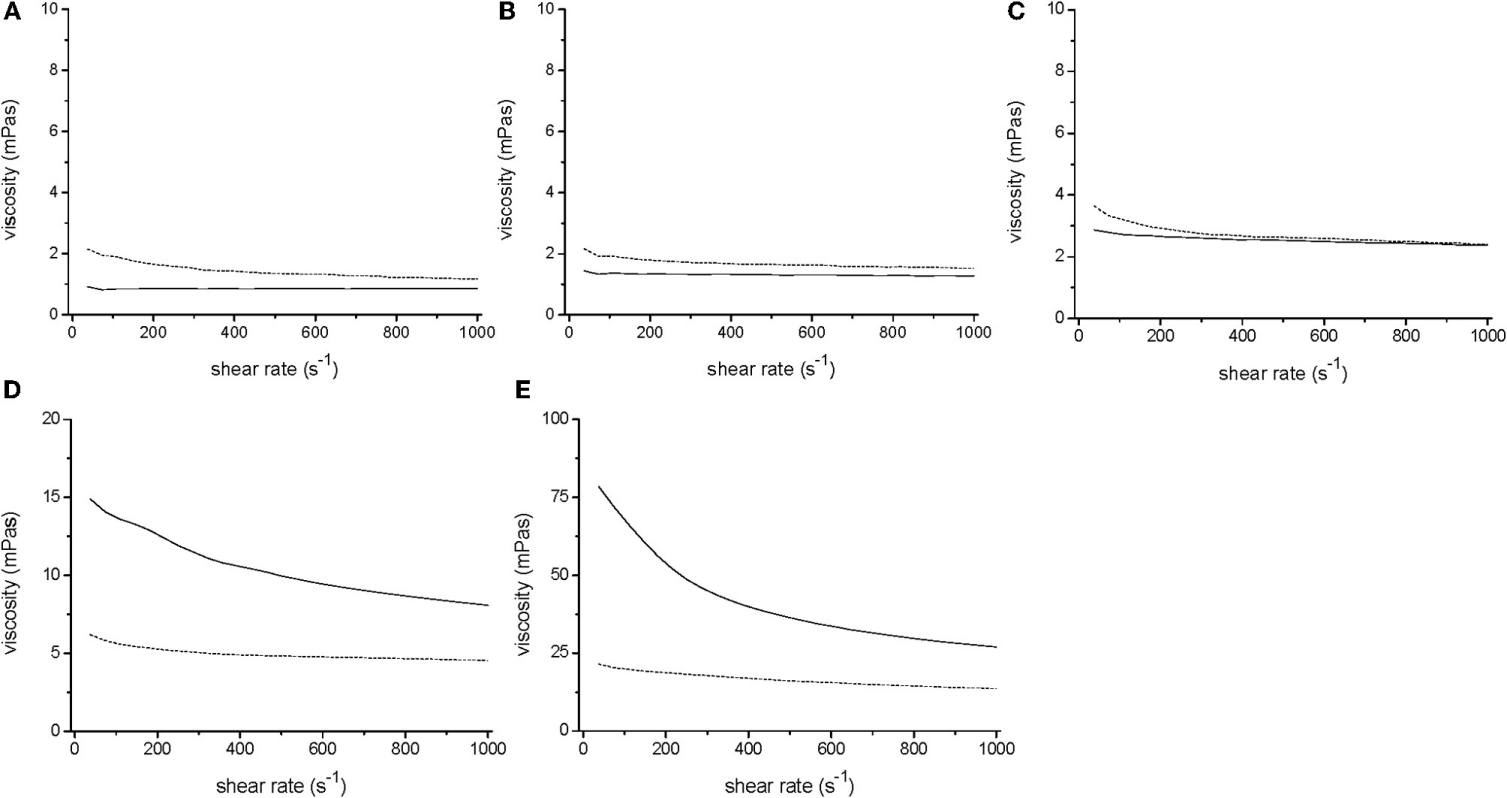
**Rheology of *V. ruber* cultures grown for 24 h (dotted line) in M9 (A), M9 with 0.1% HEC (B), M9 with 0.25% HEC (C), M9 with 0.5% HEC (D), and M9 with 1% HEC (E) together with the respective sterile media (solid line).** It should be noted that the y-axis in panels D and E have different values, i.e., 0–20 and 0–100 mPas, respectively.

During the incubation *V. ruber* changed both flow characteristics and viscosity of the growth medium (Figures 1 and 2). It increased viscosity during growth at low viscosities and significantly decreased it at high viscosities. In the M9 medium viscosity increased from 0.80 ± 0.02 to 1.3 ± 0.20 mPas during 24 h of incubation. The initial and final viscosities were equal, when the initial viscosity of the growth medium was 2.4 mPas. Above the initial viscosity of 2.4 mPas growth of *V. ruber* caused a dramatic decrease in viscosity of the medium (i.e., from 29.4 to 13.2 mPas). Vibrios are known to produce polysaccharide degrading enzymes like cellulases and chitinases (Keyhani and Roseman, 1999; Kim et al., 1999; Gao et al., 2011), which may hydrolyze HEC and cause a decrease in viscosity. Concentration of reducing sugars, as an indicator of cellulolytic activity was measured (Miller, 1959), but did not change significantly (*p* = 0.915) in M9 medium with 1% (w/V) HEC during the incubation with *V. ruber*. It was 9.0 ± 3.1 mM at the beginning and 9.2 ± 1.8 mM after 24 h of incubation. This indicates that HEC molecules were not degraded and that *V. ruber* did not exhibit cellulolytic activity. The effects of regular and thermally inactivated conditioned medium were studied as well. In both cases viscosity dropped significantly after addition of conditioned medium; i.e., from 5.2 ± 0.5 to 4.0 ± 0.3 mPas for the regular conditioned medium and from 5.7 ± 0.3 to 4.3 ± 0.1 mPas in the case of thermally inactivated conditioned medium (*p*-values 0.023 and 0.016, respectively). In all media tested *V. ruber* produced EPS. The smallest amount of EPS was produced at the lowest viscosity (7.9 × 10^−11^ mg cell^−1^), whereas at 29.4 mPas *V. ruber* produced 3.7 × 10^−10^ mg of EPS per cell. There were no statistically significant differences in EPS production at viscosities from 1.3 to 29.4 mPas. No significant change in pH or water activity upon addition of HEC was observed in the tested range (*a*_*w*_ was 0.988 ± 0.03, pH was 6.9 ± 0.1). The concentration of dissolved oxygen in sterile growth media was between 4.2 and 4.3 mg/L. During growth *V. ruber* used slightly more O_2_ at higher viscosities (Table 1). The initial O_2_ concentrations were 4.20 and 4.34, the corresponding final oxygen concentrations were 0.26 and 0.14 for 0.8 and 29.4 mPas, respectively.

**Table 1:** **Oxygen consumption, CFU values, cell size, carrying capacity, and the type of flagellum for *V. ruber* grown at different viscosities**.

**Viscosity (mPas)**	**Oxygen consumption (mg/L)**	**Final CFU/mL × 10^8^**	**Cell size (μm)**		**Carrying capacity**	**Flagellum**
			**length**	**width**		
0.8	3.92 ± 0.02	8.2 ± 0.5	2.4 ± 0.2	0.9 ± 0.1	2.13 ± 0.07	Polar
1.3	4.05 ± 0.03	6.9 ± 0.4	2.5 ± 0.1	0.9 ± 0.1	2.00 ± 0.08	Polar
2.4	4.15 ± 0.02	4.6 ± 0.9	2.4 ± 0.1	0.9 ± 0.1	2.01 ± 0.08	Polar
8.1	4.09 ± 0.01	4.8 ± 0.2	2.5 ± 0.1	0.9 ± 0.1	2.20 ± 0.05	Polar
29.4	4.20 ± 0.03	3.2 ± 0.2	2.5 ± 0.1	0.9 ± 0.1	2.10 ± 0.05	Polar

Data are shown as averages and standard deviations (n > 3).

### Growth and intracellular metabolism

**Figure 3:**
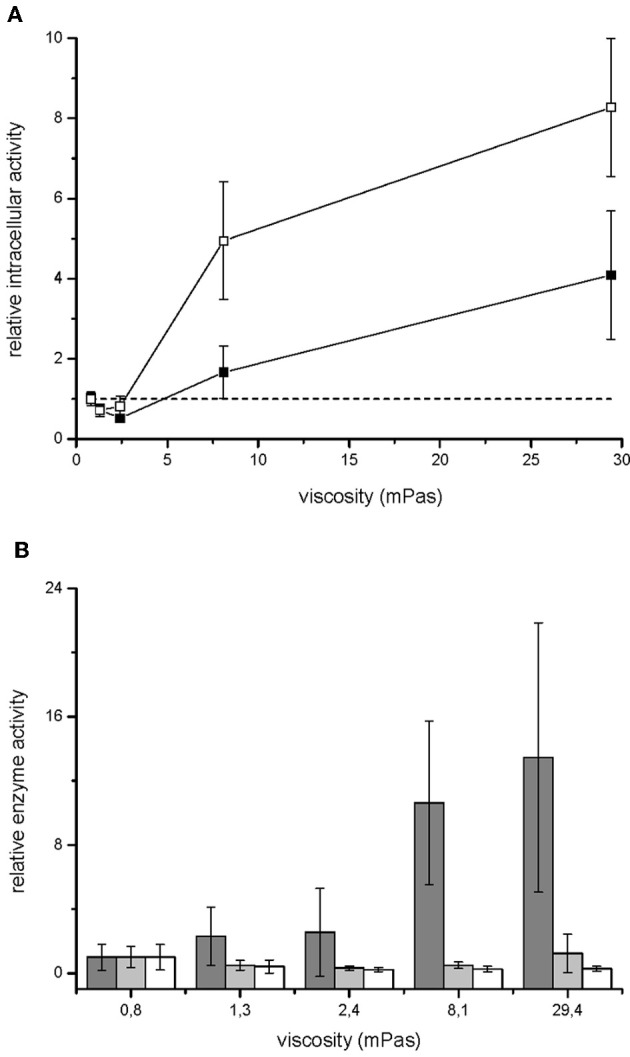
**Metabolism of *V. ruber* grown at different viscosities.** Relative respiration rate (open symbols) and dehydrogenase activity (filled symbols) of *V. ruber* are shown in panel **(A)**. The values presented are means and standard deviations (*n* = 5). The dashed line represents metabolic activity in M9 medium. Activities of glucose-6-phosphate dehydrogenase (dark gray columns), phosphofructokinase (light gray columns) and pyruvate kinase (white columns) relative to the basal enzyme activity at 0.8 mPas are shown in panel **(B)**. The values presented are means and standard deviations (*n* = 3).

Growth rate of *V. ruber* was not significantly affected (*p*-values between 0.47 and 0.96) by the viscosity of M9 growth media in the experimental range tested (Figure 1). There was also no significant change of cell size or morphology with increasing viscosity (Table 1). Bacterial motility was maintained even at the highest viscosities. There was, however, a small decrease of CFU numbers with increasing viscosity (*p*-values between 0.001 and 0.007). On the other hand, intracellular metabolic activity was substantially altered. At the highest viscosity total cell dehydrogenase activity was 1.62 ± 0.63 × 10^−1^ U mg cell protein^−1^, which is approximately four times greater than at the lowest viscosity. Total cell dehydrogenase activity correlated with respiration rate (Figure 3A). In agreement with an overall increase in dehydrogenase activity, the activity of GPD was elevated at high viscosities (Figure 3B). There was no significant difference between the highest two viscosities (*p* = 0.403). GPD activity was 1.78 × 10^−2^ U mg cell protein^−1^ at 0.8 mPas and increased to 2.40 × 10^−1^ U mg cell protein^−1^ at 29.4 mPas (*p* = 0.012). On the other hand, PFK and PK activity remained the same irrespective of the viscosity (Figure 3B) (*p*-values between 0.098 and 0.981). At the lowest viscosity, the activity of the PFK was 6.51 × 10^−3^ U mg cell protein^−1^, whereas the PK activity was 9.30 × 10^−2^ U mg cell protein^−1^.

### Secondary metabolism - prodigiosin production

**Figure 4:**
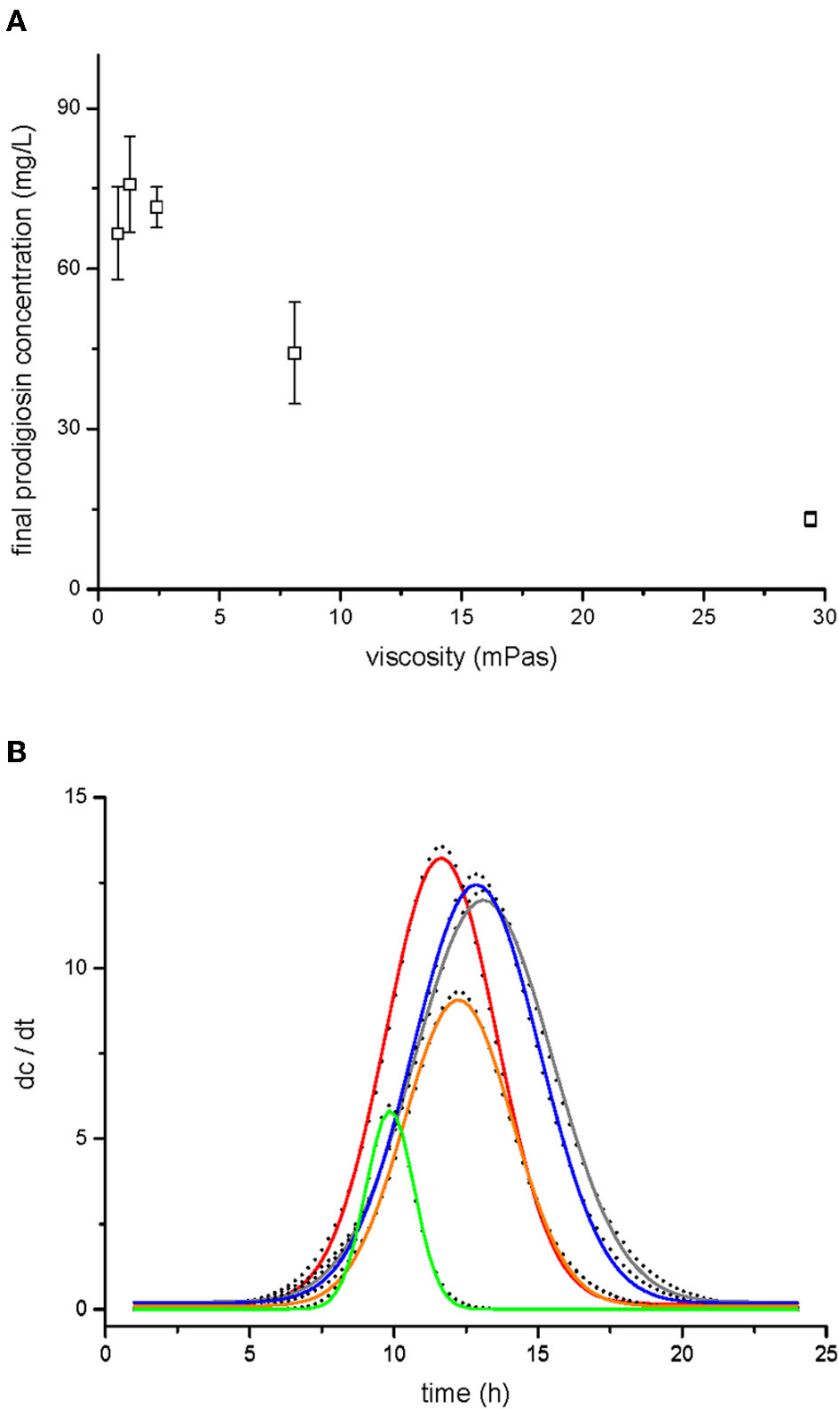
**Prodigiosin production by *V. ruber* at different viscosities.** Prodigiosin concentration after 24 h of bacterial growth is shown in panel **(A)**. The values presented are means and standard deviations (*n* = 3). Dynamics of prodigiosin production are presented in panel **(B)**. The dots represent the first derivative of prodigiosin concentration (dc/dt) and were fitted with Gaussian distribution (lines). The data presented are means of three pigment concentrations at each time of extraction. Legend: red line–0.8 mPas, gray line–1.3 mPas, blue line–2.4 mPas, orange line–8.1 mPas, green line–29.4 mPas.

Total prodigiosin production during 24 h of *V. ruber* growth at different viscosities is given in Figure 4A. When viscosity was 2.4 mPas or lower, pigment synthesis was not significantly affected (*p*-values between 0.27 and 0.48). However, at higher viscosities the production of prodigiosin decreased dramatically. For instance, at 29.4 mPas approximately five-fold less pigment was produced compared to the lowest viscosity. In addition, viscosity influenced the dynamics of prodigiosin production (Figure 4B and Table 2). At the lowest viscosity (0.8 mPas) prodigiosin synthesis started after 5 h of growth and ceased after 16 h. The maximum rate of pigment production occurred after 11.6 h of growth. With higher viscosity the onset of pigment synthesis shifted to progressively longer incubation times. For instance, at 29.4 mPas prodigiosin production started after 7.5 h of growth and declined after 12.5 h. As given in Table 2, a modest increase of viscosity from 0.8 to 1.3 mPas increased both the time when maximal prodigiosin production occurred as well as the duration of intense pigment production. With a further increase in viscosity the maximal prodigiosin production occurred earlier. Similarly, the interval of intense pigmentation first increased and then decreased with elevated viscosity.

**Table 2:** **Parameters of prodigiosin production dynamics**.

**Viscosity (mPas)**	***X*_*c*_ (h)**	***W* (h)**
0.8	11.64 ± 0.01	3.84 ± 0.02
1.3	13.11 ± 0.01	4.79 ± 0.03
2.4	12.85 ± 0.01	4.39 ± 0.03
8.1	12.24 ± 0.01	3.73 ± 0.02
29.4	9.87 ± 0.01	1.68 ± 0.01

The rate of prodigiosin production was approximated with Gaussian distribution (R^2^ > 0.998). W is the width of the Gaussian curve and X_c_ is the maximal value of the Gaussian curve. The values presented are means and standard deviations (n = 3).

X_c_ represents the time of maximal pigment production.

W represents the duration of intense pigment production.

## Discussion

Viscosity is an ever present property of the environment that influences many important microbial processes, namely molecular diffusion and transport. It contributes substantially to the observed differences in the physiological state of planktonic and biofilm cells (Davey and O'Toole, 2000; Walters et al., 2003; Fux et al., 2005; Resch et al., 2005, 2006; Spormann, 2008; Stewart and Franklin, 2008). Additionally, viscosity has a role in bacterial motility, virulence, cooperation (Kümmerli et al., 2009; Le Gac and Doebeli, 2010), and antimicrobial resistance (Kostenko et al., 2007). There is, however, no systematic study of the impact of viscosity on bacterial metabolic activity.

Cellulose and its derivatives are abundant polymers that are used to increase viscosity (Shigematsu et al., 1998). HEC is used in different industries as a thickening agent and was used in this study to change the viscosity of the minimal M9 growth medium. It is important to note that HEC is neither used by *V. ruber* as the sole carbon source nor is it enzymatically degraded. To check for physico-chemical changes in the growth medium we have measured oxygen consumption, pH, and water activity upon HEC addition. None of them were significantly altered with the addition of HEC. Viscosities studied in this work range from 0.8 to 29.4 mPas (measured at shear reate of 1000 s^−1^) and represent environments of intermediate viscosity. For instance, distilled water has the viscosity of 0.8 mPas at 20°C, while viscosity of sea water can be between 0.8 and 1.8 mPas depending on the temperature (Fofonoff, 1962). Viscosity of biofilms, on the other hand, ranges from 1 Pas to as high as 10^8^ Pas (Hall-Stoodley et al., 2004; Cheong et al., 2009). Viscosity of human mucus, saliva or tears is approximately 100-fold higher than water at low shear stress and becoming less viscous at higher shear stress (Cone, 1999; Lai et al., 2009).

The most important result of this work is that viscosity of the growth medium changes both the primary and secondary metabolism of *Vibrio ruber*. Cells were stressed at higher viscosities and increased respiration rate (Figure 3A). It is expected that the increased CO_2_ production rates at higher viscosities are mainly the result of increased dehydrogenase activity (Roy and Packard, 2001; Créach et al., 2003; Danevčič and Stopar, 2011). The total dehydrogenase activity is a global physiological parameter that depends on the rate of microbial metabolism in glycolysis, citric acid cycle, and electron transport chain. The flow of carbon through glycolysis was determined with PFK activity. The entrance of carbon into the citric acid cycle was monitored with PK activity whereas the carbon flow through pentose phosphate pathway was determined with GPD activity. While activities of PFK and PK did not change at different viscosities, GPD was significantly increased at higher viscosities (Figure 3B). A similar stress response of *V. ruber* was observed at extreme salinities (Danevčič and Stopar, 2011). Although both GPD and total dehydrogenase activities increased with viscosity the GPD increased 13 fold compared to 4 fold increase in total dehydrogenase activity. It must be pointed out that total dehydrogenase activity and GPD activity were measured with different methods, and therefore the results are not directly comparable. Furthermore, glucose-6P-dehydrogenase is a specially positioned enzyme in the metabolic network that acts as a shunt to the pentose phosphate pathway. The pentose phosphate pathway provides reducing equivalents and carbon intermediates for biosynthesis. Although carbon from pentose phosphate pathway may end up in the citric acid cycle and therefore contribute to the total dehydrogenase activity, it can also enter other metabolic pathways. The observed difference between total dehydrogenase activity and glucose-6P-dehydrogenase may therefore indicate a change in regulation at the level of pentose phosphate pathway at different viscosities. An increased flow of carbon through the pentose phosphate pathway may also explain why increased CO_2_ production rate was observed in the absence of significant change of flow through glycolysis and the citric acid cycle at high viscosities.

Prodigiosin is a secondary metabolite with a molecular mass of 323 g mol^−1^ (Borić et al., 2011). Its synthesis is controlled by quorum sensing (Thomson et al., 2000; Danevčič and Stopar, 2009), a process that is likely to be affected by environmental viscosity. Increased viscosity did not change only the onset of pigment production but also the overall dynamics of prodigiosin synthesis (Figure 4B, Table 2). At low viscosities prodigiosin synthesis started earlier and lasted longer, allowing production of larger quantities of pigment (Figure 4A). At the highest viscosity, on the other hand, pigmentation occurred in a short burst leading to smaller amount of prodigiosin. The varying duration of intense pigmentation suggests that in addition to quorum sensing other regulatory elements control pigment synthesis. Prodigiosin synthesis requires precursors like L-proline, acetate, L-serine, S-adenosylmethionine, and 2-octenal (Quadri and Williams, 1973; Wasserman et al., 1973; Williamson et al., 2006). Since none are present in the M9 medium, *V. ruber* must synthesise them *de novo*. Pigment biosynthesis also requires copious amount of redox equivalents (Trutko and Akimenko, 1989). This makes prodigiosin synthesis costly and in co-cultures non-pigmented mutants overgrow pigmented cells (Borić et al., 2011). In order to avoid high energetic costs prodigiosin synthesis can be easily interrupted in unfavourable conditions (Williamson et al., 2006; Starič et al., 2010). Our results indicate that *V. ruber* reduces prodigiosin synthesis at higher viscosities.

It is interesting to note that *V. ruber* decreased pigment production and reduced CFU, but maintained high GPD activity in more viscous media. This suggests increased activity of pentose phosphate pathway, which provides reduction equivalents and metabolic intermediates. Increased metabolic flux through pentose phosphate pathway at higher viscosities may be used for EPS biosynthesis or locomotion. Approximately five-fold increase in EPS production was indeed observed at 2.4 mPas. However, at viscosities between 2.4 and 29.4 mPas, where cells significantly increased metabolic rate, the EPS production did not change significantly. It is therefore possible that increased metabolic activity may be used for locomotion, which gets excessively difficult at higher viscosities as bacterial flagellae experience a dampening action by viscous media (Schneider and Doetsch, 1974; Ferrero and Lee, 1988; Shigematsu et al., 1998). It has been extensively confirmed that many morphologically different bacteria increase swimming speed with small increase in viscosity of the medium (up to 2 mPas for polarly flagellated bacteria). With further increase in viscosity swimming speed decreased. Our results consistently indicate a major shift in metabolic activity above 2.5 mPas. Some bacteria, including two *Vibrio* species, change from polar to lateral flagellation at increased viscosity (Schneider and Doetsch, 1974; Greenberg and Canale-Parola, 1977; Belas et al., 1986; Ferrero and Lee, 1988; Atsumi et al., 1996). Examination with TEM showed that *V. ruber* remained polarly flagellated at all studied viscosities (Table 1) and did not cease moving at high viscosities. Movement of polar flagellum is a large energy sink for a bacterium (Atsumi et al., 1992; Kojima et al., 1999) and being able to move is essential for providing nutrient flow into cells. There is a radius around cells, in which diffusion still provides the cells with needed nutrients. However, when nutrients are depleted, cells are forced to move somewhere else. Since viscosity increased exponentially with HEC addition, it is likely that energetic cost for mobility of *V. ruber* at higher viscosities also increased substantially.

*V. ruber* has the ability to change the viscosity of its environment (Figures 1, 2). A small increase in viscosity below 2.4 mPas may be attributed to EPS production. It is, however, remarkable that cells produced more EPS at 8.1 mPas, but were nevertheless able to significantly reduce overall viscosity of the medium. The results further suggest that observed decrease in viscosity could not be correlated with cellulolytic enzyme activity. At present it is not known what may cause such a dramatic decrease in viscosity. Amino acids, metal ions and simple sugars have been implied in altering viscosity of polysaccharide solutions (Parker et al., 1996; Mazurkiewitz et al., 2001; Tang et al., 2005). Other chemical compounds like chlorides, chelates, and urea have been shown to modulate biofilm viscosity (Chen and Stewart, 2002). Some of theses molecules might be present in the conditioned medium and cause the observed drop in viscosity. Furthermore, it is possible that bacteria struggling to move at higher viscosities may reduce viscosity of bacterial growth medium (Sokolov and Aranson, 2009). The ability of bacterial species to change viscosity of their environment is not well understood.

In conclusion, it is noteworthy that cells maintain homeostasis (i.e., the same growth rate) in spite of 37-fold increase of viscosity. In order to do so, *V. ruber* increases its central metabolism at higher viscosities and at the same time reduces biosynthesis of secondary metabolite prodigiosin. Both triggering and regulation of prodigiosin synthesis depend on viscosity. In addition, *V. ruber* influences the viscosity of its environment. Our results raise several important and interesting questions such as what are the molecular mechanism underlying the observed response as well as how widespread such a response is in other bacteria that need to be further investigated.

### Conflict of interest statement

The authors declare that the research was conducted in the absence of any commercial or financial relationships that could be construed as a potential conflict of interest.

## Appendix

### Bacterial strain, gene sequencing and phylogenetic analysis

Genomic DNA was extracted from *Vibrio* sp. DSM 14379 using GenElute Bacterial Genomic DNA Kit (Sigma, USA). Genomic DNA was sequenced using paired-end methodology on the Illumina GAII analyzer (Illumina, Inc., San Diego, USA) by BaseClear (Leiden, The Netherlands). The reads were assembled with Edena v.2.1.1 software (Hernandez et al., 2008). To construct phylogenetic tree 15 closely related *Vibrio* type strains with 7 gene loci (16S *rRNA*, *rpoA*, *recA*, *ftsZ*, *gapA*, *gyrB* and *mre*B) were chosen. The accession numbers for the sequences used in MLSA (Multilocus Sequence Analysis) to construct the phylogenetic tree are shown in Table A1. MLSA were based on the concatenated nucleotide sequences of the selected genes, which were aligned by ClustalW (Higgins et al., 1996). The phylogenetic analyses were conducted using MEGA version 5.0 (Tamura et al., 2011). The phylogenetic interference was based on the Maximum Parsimony method (MP). The robustness of the topology was checked by 1000 bootstrap replications. The obtained phylogenetic tree is shown in Figure A1.

**Table A1:** **Bacterial strains and accession numbers for gene sequences used in the MLSA**.

	**16S rRNA gene**	***rpoA* gene**	***recA* gene**	***ftsZ* gene**	***gapA* gene**	***gyrB* gene**	***mreB* gene**
*Vibrio ruber* DSM 14379	EU735067	JQ582880	JQ582881	JQ582885	JQ582884	JQ582883	JQ582882
*Vibrio gazogenes* ATCC 29988	X74705	AJ842616	AJ842429	DQ907348	DQ907284	AB298258	DQ907420
*Vibrio ruber* JCM 11486	AF462458	EF523235	EF523236	DQ907373	DQ907306	AB298267	EF114211
*Vibrio mangrovi* LMG 24290	EU144014	EU144015	EU144016	FJ876001	EU713844	EU713845	GU259617
*Vibrio furnissii* ATCC 35016	X76336	AJ842614	AJ842427	EF027345	DQ907283	AB298217	DQ907418
*Vibrio fluvialis* NCTC 11327	X76335	AJ842606	AJ580892	DQ907345	DQ907281	AB298215	DQ907416
*Vibrio mimicus* ATCC 33653	X74713	AJ842653	AJ842468	DQ907357	DQ907292	EU680783	DQ907430
*Vibrio campbellii* ATCC 25920	X56575	AJ842564	AJ842377	DQ907337	DQ449614	EU130500	DQ907408
*Vibrio orientalis* ATCC 33934	X74719	AJ842672	AJ842485	DQ907365	DQ907299	EF380260	DQ907439
*Vibrio alginolyticus* ATCC 17749	X56576	AJ842558	AJ842373	EF027344	DQ907274	EU680781	DQ907405
*Vibrio brasiliensis* LMG 20546	AJ316172	AJ842563	AJ842376	DQ907335	DQ449613	AB298204	HM771369
*Vibrio coralliilyticus* LMG 20984	AJ440005	AJ842587	AJ842402	DQ907341	DQ907279	AB298210	DQ907412
*Vibrio harveyi* ATCC 14126	AB680920	AJ842627	DQ648369	DQ907350	EF596145	EF596215	DQ907422
*Vibrio natriegens* ATCC 14048	X74714	AJ842658	AJ842473	DQ907359	DQ907294	AB298232	JF930490
*Vibrio splendidus* LMG 4042	AJ515230	AJ842725	AJ842511	DQ481635	DQ481622	EF380261	DQ481647
*Vibrio vulnificus *ATCC 27562	X76333	AJ842737	AJ842523	DQ520272	DQ907313	GQ382199	DQ907454
